# Albumin Paclitaxel Combined with Intrapleural Infusion of Bevacizumab + Lobaplatin for the Second-Line Treatment of Patients with Non-Squamous Non-Small Cell Lung Cancer

**DOI:** 10.1155/2022/5901450

**Published:** 2022-06-26

**Authors:** Junjie Hou, Xuguang Mi, Ning Liu, Ying Yang, Zhaoxue Qi, Xiaonan Li, Xiaodan Lu, Xianzhuo Jiang, Yingying Yu, Ying Zhou, Zhiqiang Ni, Yanqiu Fang, Ningyi Jin

**Affiliations:** ^1^Medical College, Yanbian University, Yanji, China; ^2^Department of Comprehensive Oncology, Jilin Province People's Hospital, Changchun, China; ^3^General Surgery of the First Clinical Hospital of Jilin Academy of Chinese Medicine Sciences, Changchun, China; ^4^Department of Secretory Metabolism, The First Hospital of Jilin University, Changchun, China; ^5^Changchun Institute of Veterinary Medicine, Chinese Academy of Agricultural Sciences, Changchun, China

## Abstract

**Objective:**

To investigate the clinical efficacy and safety of albumin paclitaxel combined with intrapleural bevacizumab + lobaplatin for patients with non-squamous non-small cell lung cancer (NS-NSCLC) with malignant pleural effusion (MPE) and analyze prognostic factors.

**Methods:**

A total of 126 NS-NSCLC patients were included in the study. Control group with 64 cases received intrapleural infusion of lobaplatin + intravenous albumin paclitaxel, and treatment group with 62 cases received additional intrapleural bevacizumab perfusion. Analysis was performed by collecting data about MPE, progression-free survival (PFS), overall survival (OS), and scores of quality of life.

**Results:**

In the treatment and control groups, objective response rate (ORR) was 51.6% and 31.3% (*χ*^2^ = 5.39, *P*=0.02), and disease control rate (DCR) was 91.9% and 71.9% (*χ*^2^ = 8.49, *P*=0.004), respectively. The main adverse reactions (≥grade 3) in the treatment group were thrombocytopenia, peripheral neurotoxicity, proteinuria, neutropenia, and nausea/vomiting, and in the control group, they were weakness, nausea/vomiting, anemia, and peripheral neurotoxicity. In the control and treatment groups, the median PFS was 6.2 (95% confidence interval (CI): 5.86–6.56) and 5.1 (95% CI: 4.956–5.191), and the median OS was 14.4 (95% CI: 12.681–16.113) and 10.6 months (95% CI: 8.759–12.391). The score of quality of life for treated patients was significantly higher than those before treatment and the control group, and the parameters included general health status (GH), role physical (RP), body pain (BP), social function (SF), and vitality (VT); pH, CD4+/CD8+ values, and vascular endothelial growth factor (VEGF) in the pleural effusion significantly affected the PFS and OS (*P* < 0.05). Bevacizumab administration in patients with bloody pleural effusion did not increase the risk of pleural hemorrhage.

**Conclusion:**

The combination of albumin paclitaxel and intrapleural bevacizumab + lobaplatin is effective and may reverse the adverse events in patients with NS-NSCLC and MPE. The change of CD4+/CD8+ ratio before and after treatment is an independent and prognostic factor for patients with NS-NSCLC and MPE.

## 1. Introduction

Lung cancer is one of the most common malignancies. According to the pathological type, it can be divided into small cell lung cancer (SCLC) and non-small cell lung cancer (NSCLC), and the ratio of NSCLC is as high as 80%, in which non-squamous non-small cell lung cancer (NS-NSCLC) accounts for the vast majority of NSCLC. The growth and metastasis of NSCLC are slower than those of SCLC [1]. Research [2] showed that about 75% of NSCLC patients were in the advanced stage at admission, and the best opportunity for surgery was missed. Chemotherapy is usually recommended, and platinum-based dual-drug combination chemotherapy is a common treatment plan for patients with advanced NSCLC. Chemotherapy has problems such as poor sensitivity, unsatisfactory curative effects, easy recurrence, and metastasis [3]. To improve the survival rate of patients with advanced NSCLC, it is of great significance to find new treatment methods.

A recent research [4] found that the growth of tumor tissues relied on neovascularization, which provided sufficient oxygen and nutrients for tumor cells and is closely related to tumor occurrence, development, invasion, and metastasis. At present, among the known angiogenesis-promoting factors, vascular endothelial growth factor (VEGF) has the most significant effect. It is closely related to tumor angiogenesis and has the highest stability and the strongest function. The changes in VEGF levels can reflect tumor cell activity [5]. Bevacizumab is the first VEGF inhibitor approved for marketing in the world. Pharmacological studies [6] have shown that bevacizumab competitively binds to VEGF to inhibit the division and proliferation of vascular endothelial cells and, at the same time, increase vascular permeability, thereby blocking tumor angiogenesis and ultimately exerting an antitumor effect.

Malignant pleural effusion (MPE) is a common and refractory complication of advanced NS-NSCLC, especially in patients who relapse after first-line treatment [7, 8]. The MPE visually appears bloody or turbid amber. Traditional treatment techniques for MPE include intracavity infusion, chemotherapy, biological therapy, puncture, drainage, and others [9–11]. Although they have been widely used in clinical practice, the curative effect is not satisfactory because patients with such relapses are usually in poor general condition and poorly tolerated to treatment. The level of VEGF in pleural effusion was significantly higher than that in serum. The VEGF levels in plasma and pleural effusion can predict outcome of bevacizumab treatment in patients with NSCLC and MPE [12–14]. Since several clinical studies have confirmed the important role of VEGF in the pathogenesis of MPE, intrapleural infusion of the antiangiogenic drug bevacizumab has become a new clinical treatment method for malignant pleural effusion. However, most studies focused on the efficacy and adverse reactions of intrapleural infusion of bevacizumab and chemotherapeutic drugs such as cisplatin and paclitaxel. There was no research reported on the intravenous administration of albumin paclitaxel combined with a local intrapleural infusion of bevacizumab and lobaplatin for a single cancer type.

This study attempts to investigate the clinical efficacy and safety of albumin paclitaxel combined with intrapleural infusion of bevacizumab + lobaplatin for the second-line treatment of driver gene-negative NS-NSCLC patients with MPE and analyze the factors affecting the prognosis. We focus on observing and recording changes in MPE, progression-free survival (PFS), overall survival (OS), and quality of life of patients. This study will provide data and new treatment plans to support clinical treatment for patients with driver gene-negative NS-NSCLC patients with MPE.

## 2. Materials and Methods

### 2.1. Clinical Data

A total of 126 NS-NSCLC patients who were admitted to the People's Hospital of Jilin Province from March 2019 to December 2020 were selected as the research subjects. According to different treatment methods, they were divided into the control group with 64 cases (intrapleural infusion of lobaplatin + intravenous albumin paclitaxel) and a treatment group with 62 cases (combined with intrapleural bevacizumab perfusion therapy in addition to the treatment of the control group). Both groups of patients were lung adenocarcinoma without driver gene mutations. This study was approved by the Ethics Committee of Jilin Provincial People's Hospital, and the patients and their families had signed informed consent forms. The age range of the two groups of patients was 37–75 years old. The gender ratio was 3 : 2 with more male patients than females, and the stage of cancer was stage IV (M1a-M1c): control group (51 cases of stage M1a; 7 cases of stage M1b; 6 cases of stage M1c); treatment group (49 cases of stage M1a; 8 cases of stage M1b; 5 cases of M1c). The first-line treatment plan received by the two groups of patients: (Pemetrexed + cisplatin) for 47 cases in the control group and 50 cases in the treatment group; (paclitaxel + cisplatin) for 12 cases in the control group and 8 cases in the treatment group; (Gemcitabine + Cisplatin) for 5 cases in the control group and 4 cases in the treatment group. Baseline data such as age, gender, tumor stage, bloody and non-bloody plural effusion, VEGF expression levels, pleural effusion pH value, and pleural effusion CD4+/CD8+ ratio between the two groups of patients were compared. There were no statistical differences (*P* < 0.05) and were comparable (Table 1).

Inclusion criteria: (1) patients meet the relevant diagnostic criteria for NS-NSCLC [15] and have been confirmed by pathology or cytology; (2) patients diagnosed as NS-NSCLC stage IV by histopathology, and genetic testing showed no driver gene (EGFR, ALK) mutations; (3) MPE is confirmed by identifying malignant cells in pleural effusion caused by tumor metastasis in the lung; (4) NS-NSCLC patients who have failed first-line treatment and have complete clinical data; (5) patients who are unable to accept surgery or refuse to undergo surgery, with a Karnofsky score ≥70 points; (6) patients with the expected survival time >3 months and those who meet the indications for chemotherapy.

Exclusion criteria: (1) patients with small cell lung cancer or squamous NSCLC or driver gene (EGFR, ALK) mutations; (2) patients with severe diseases of the heart, liver, kidney, and other important organs; (3) patients with other malignant tumors; (4) patients with cognitive dysfunction who cannot cooperate with treatment; (5) pregnant and lactating patients.

### 2.2. Treatment Methods

According to the difference in the combination of bevacizumab, 126 cases of NS-NSCLC patients were randomly divided into two groups: the treatment plan of the control group was intrapleural infusion of lobaplatin (Hainan Changan International Pharmaceutical Co., Ltd. Patentee: ASTA Pharmaceutical Corporation; Patent No.: ZL94106670.3) (30 mg/2 weeks) + intravenous albumin paclitaxel (China Shijiazhuang Pharmaceutical Group, Ouyi Pharmaceutical Co., Ltd.) (single week plan, 100 mg/week), and the treatment plan of the treatment group was intrapleural infusion of lobaplatin (30 mg/2 weeks) + bevacizumab (commodity name: Avastin, a product of Shanghai Roche Company; Patentee: California Genentech Corporation, USA; Patent No.: US 6,884,879 B1) (300 mg/2 weeks) + intravenous albumin paclitaxel (100 mg/week for one-week regimen). The drug withdrawal criteria are CR, ineffective or intolerable toxicity.

In order to ensure that repeat thoracic drainage and repeated and regular intrathoracic administration of drugs in the patients can be performed, a pigtail catheter is placed in the patient's thoracic cavity (12 Ga) (ABLE, Foshan Nanhai Lily Medical Technology Co., Ltd.). All procedures were done with the guidance of Color Doppler ultrasound (most of the puncture points were taken from the 7th or 8th intercostal axillary front or posterior axillary line of the diseased side). Salient technical aspects of pigtail catheter insertion included appropriate use of local anesthetic and needle insertion to avoid the intercostal bundle. We typically employed a small needle (16 Ga) before inserting the larger one provided with the kit. In this way, pleural fluid was easily withdrawn with the needle, and passage of the guidewire was effortlessly put into the pleural cavity. Finally, the catheter was retained to a depth of 15 cm in the pleural cavity. There are 6 side holes at the distal end of the catheter. With an adequate insertion of the pigtail, the side holes were well within the pleural cavity for proper function. The pigtail catheter was attached to a standard thoracic drainage system, and suction was applied for treatment of pleural fluid.

### 2.3. Observation Indicators and Evaluation Criteria

#### 2.3.1. Efficacy Evaluation

The treatment efficacy was evaluated after every 2 cycles of chemotherapy and rechecked every 1 month after the end of chemotherapy. Efficacy evaluation was based on previous studies [16] and recorded as Complete response (CR), partial response (PR), stable disease (SD), and progressive disease (PD). The objective response rate (ORR) was the ratio of CR + PR, and the disease control rate (DCR) was the ratio of CR + PR + SD.

#### 2.3.2. Immune Function Indicators

The pleural effusion of the two groups of patients was collected in the morning before treatment and the first day after the end of the treatment course. The flow cytometry and its supporting reagents were used, and the operating instructions were strictly followed to complete the detection of relevant indicators of *T* cell subsets within 6 hours. The CD4^+^/CD8^+^ ration of the two groups of patients was detected and recorded in detail.

#### 2.3.3. Cell Growth Factor Determination

The pleural effusion of the two groups of patients was collected, and the enzyme-linked immunosorbent assay was used to detect VEGF.

#### 2.3.4. Survival Analysis

The patient follow-up was conducted by telephone or revisits. PFS is the time from the date of first treatment to disease progression or death due to any reason. OS is the time from the patient's first treatment to death. Those who were lost to follow-up were considered censored. PFS was calculated as the time to the last effective evaluation, and OS was calculated as the time to the last follow-up.

#### 2.3.5. Adverse Reactions

Common Terminology Criteria for Adverse Events version 4.0 (CTCAE 4.0) were used for the evaluation of adverse events.

#### 2.3.6. Quality of Life Score

The MOS item short from the health survey (SF-36) was used for evaluation, and the general health status (GH), role physical (RP), body pain (BP), social function (SF), vitality (VT), and other dimensions were used for evaluating the quality of life of patients. To facilitate comparative research, the standard scores were unified into a 100-point system. The higher the score, the higher the quality of life of the patient.

### 2.4. Statistical Analysis

SPSS 25.0 was used for analysis. The clinical efficacy, clinical-pathological characteristics, and adverse reactions of the patients were compared by the *χ*^2^ test. The quality of life score was compared by the *t*-test. The Kaplan-Meier method was used for survival analysis. The Log-rank test was used for univariate analysis. The Cox regression model was used for multivariate analysis. *P* < 0.05 indicated that the difference was statistically significant.

## 3. Results

### 3.1. Comparison of Clinical Efficacy between the Two Groups of Patients

**Figure 1:**
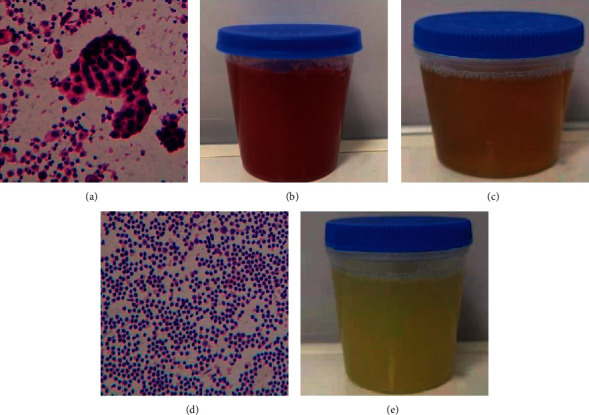
Pathological image. (a)–(c), before treatment. (a) Image of pleural effusion under the microscope; (b) hemorrhagic MPE; (c) turbid amber MPE; (d) image of pleural effusion under microscope of patients with effective treatment; (e) MPE (color tends to clear) in a patient with effective treatment.

All 126 patients were evaluated for treatment efficacy. In the treatment group, there were 4 cases of CR, 28 cases of PR, 25 cases of SD, and 5 cases of PD. In the control group, there were 0 cases of CR, 20 cases of PR, 26 cases of SD, and 18 cases of PD. The ORR of the two groups was 51.6% and 31.3% (*χ*^2^ = 5.39, *P*=0.02), and the DCR of the two groups was 91.9% and 71.9% (*χ*^2^ = 8.49, *P*=0.004), respectively. The differences were statistically significant (*P* < 0.05, Table 2). The color of the pleural effusion of patients with effective treatment became lighter and tended to be clear, the tumor cells in the pleural effusion under the microscope were reduced or disappeared, and the number of lymphocytes increased (Figure 1).

### 3.2. Comparison of Adverse Reactions between the Two Groups of Patients

Adverse reactions of all patients were evaluated. The main adverse reactions of the treatment group with ≥grade 3 reactions were as follows: hypertension, neutropenia, proteinuria, thrombocytopenia, fatigue, nausea/vomiting, anemia, and peripheral neurotoxicity. The main adverse reactions of the control group with ≥grade 3 reactions were fatigue, nausea/vomiting, anemia, and peripheral neurotoxicity. There were no treatment-related deaths in the two groups. There was no statistical difference in adverse reactions between the two groups (*P* > 0.05, Table 3).

### 3.3. Survival Analysis of the Two Groups of Patients

**Figure 2:**
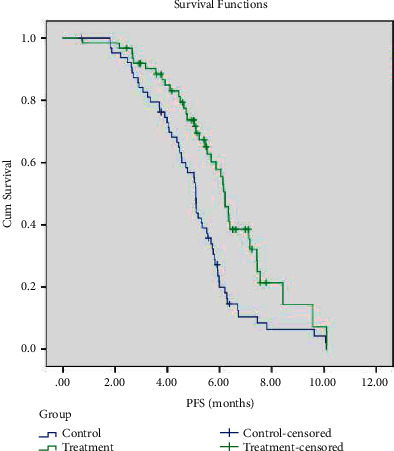
PFS survival curves of the two groups of patients.

**Figure 3:**
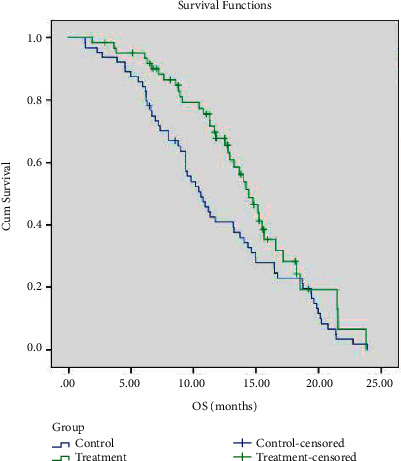
OS survival curves of the two groups of patients.

The last follow-up time was June 31, 2021. The follow-up duration was 1–25 months. The median PFS of the treatment group and the control group was 6.2 months (95% CI: 5.86–6.56 months) and 5.1 months (95% CI: 4.956–5.191 months), respectively; the difference was statistically significant (*P*=0.001, Figure 2). The median OS of the treatment group and the control group was 14.4 months (95% CI: 12.681–16.113 months) and 10.6 months (95% CI: 8.759–12.391 months), respectively; the difference was statistically significant (*P*=0.039, Figure 3).

### 3.4. Comparison of Quality of Life Scores between the Two Groups

Before treatment, there was no statistically significant difference in the quality of life scores between the two groups of patients (*P* > 0.05). After 3 months of treatment, the quality of life scores of patients in the treatment group was significantly improved in the dimensions of GH, RP, BP, SF, and VT, and the scores were higher than those of the control group. The quality of life scores of the patients in the control group was improved in the dimensions of RP and BP compared with those before treatment, and the difference was statistically significant (*P* < 0.05, Table 4).

### 3.5. Univariate Analysis

**Figure 4:**
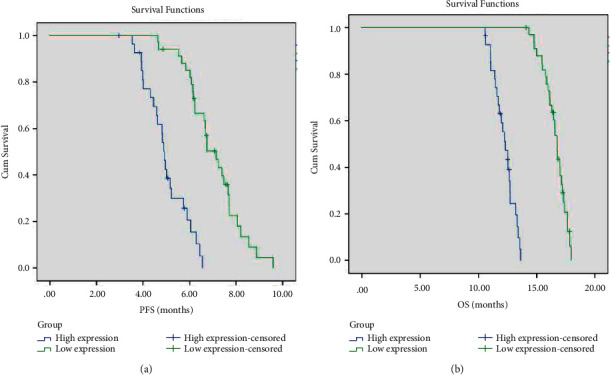
PFS (a) and OS (b) survival curves of patients with different VEGF expression levels in the treatment group.

**Figure 5:**
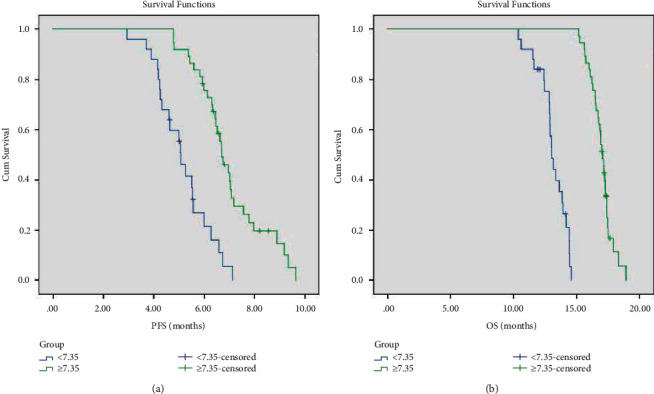
PFS (a) and OS (b) survival curves of patients with different pleural effusion PH values in the treatment group.

**Figure 6:**
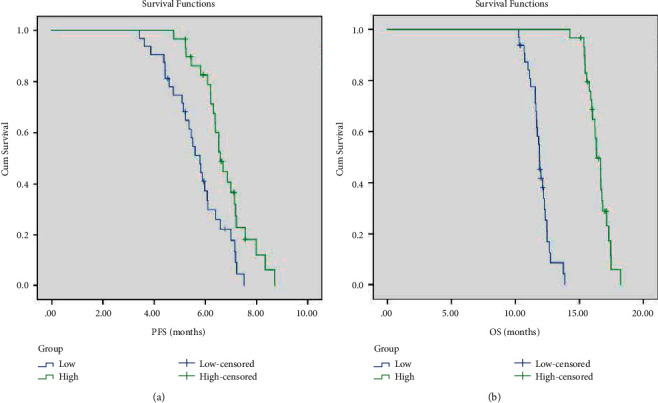
PFS (a) and OS (b) survival curves of patients with different pleural effusion CD4^+^/CD8^+^ ratios in the treatment group.

**Figure 7:**
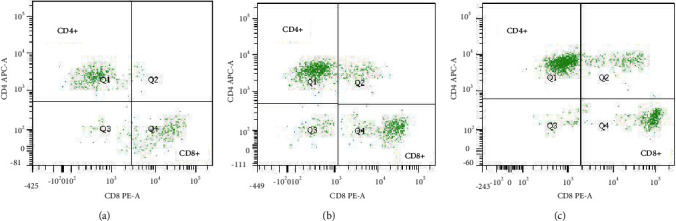
Flow cytometric analysis on changes in the immune function of patients with better prognosis in the treatment group (it represents the change of CD4/CD8 ratio during the effective treatment of patients, and the ratio is getting higher and higher. Three continuous flow charts of typical patients were used here).

**Figure 8:**
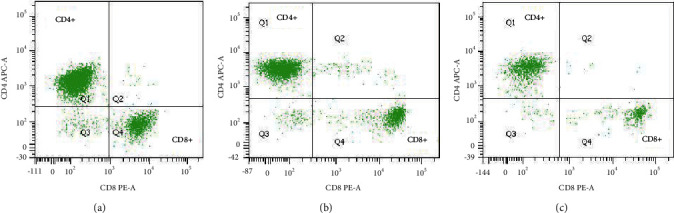
Flow cytometric analysis on the immune function of patients with poor prognosis in the treatment group (it represents the change of CD4/CD8 ratio in patients with poor therapeutic efficacy during treatment, with the ratio getting lower and lower. Three continuous flow charts of typical patients were used here).

The clinicopathological factors of the patients in the treatment group were included in the univariate analysis. The results showed that patients with low expression of VEGF, pleural effusion pH ≥ 7.35, and high CD4^+^/CD8^+^ ratio in pleural effusion had a longer survival period and more effective treatment (Table 5, Figures 4–6). As shown in the flow cytometric analysis on the immune function changes in patients with good prognosis in the treatment group, Figure 7(a) was the initial stage of treatment, and the CD4^+^/CD8^+^ ratio was low; Figure 7(c) had the highest CD4^+^/CD8^+^ ratio.  Figure 8 was a flow cytometric analysis on immune function changes in patients with poor prognosis in the treatment group, and the CD4^+^/CD8^+^ ratio gradually decreased.

### 3.6. Multivariate Analysis

The statistically significant indicators in the univariate analysis were subjected to Cox regression multivariate analysis. The results showed that VEGF and pH in pleural effusion before treatment were independent poor prognostic factors, and the trend of CD4^+^/CD8^+^ changes in pleural effusion before and after treatment was an independent predictor of treatment efficacy.

## 4. Discussions

In recent years, the incidence of NS-NSCLC has been increasing [17]. The main clinical symptoms of patients are bloody sputum, cough, weight loss, chest pain, and dyspnea, which seriously affect the quality of life of patients. Most advanced NS-NSCLC cannot be surgically removed, and the 5-year survival rate is low, ranging from 4% to 17% [18]. At present, there is a lack of specific molecular targeted drugs for the treatment of advanced NS-NSCLC patients with negative driver genes in clinical practice. Stereotactic radiotherapy, three-dimensional conformal radiotherapy, combined with platinum, and other concurrent radiotherapy and chemotherapy are the main clinical treatments, but the survival rate is not improved. Therefore, it is particularly important to find effective drugs to improve the survival rate of advanced non-squamous and non-small cell lung cancer with negative driver genes. Bevacizumab is a recombinant humanized monoclonal antibody. As an angiogenesis blocker, it competitively binds to the vascular endothelial growth factor released by cancer tissues, blocks angiogenesis in cancer tissues, and inhibits tumor cell growth. Moreover, it can improve the structure, function, microenvironment, and other abnormal blood vessels, promote the penetration of chemotherapeutic drugs to reach the cancer tissues, and exert the anti-cancer effect. The special targeting of bevacizumab, on the one hand, can cut off the blood nutritional supply channel of cancer tissues and, on the other hand, can dredge the “blood vessels” and assist other anti-cancer drugs to concentrate on cancer tissues [19]. The efficacy and safety of bevacizumab combined with chemotherapy for newly treated NS-NSCLC patients had been confirmed by several studies [20, 21], but the efficacy of this combination therapy program for the second-line and above treatment has not yet been confirmed [22, 23]. Therefore, this study intended to explore the clinical efficacy and safety of albumin paclitaxel combined with intrapleural infusion of bevacizumab + lobaplatin for the second-line treatment of patients with driver gene-negative NS-NSCLC with pleural effusion and analyze the factors that affect the prognosis.

Nishino et al. [24] reported that 45 patients in two groups received second-line treatment with docetaxel plus bevacizumab and tegafur, gimeracil, and oteracil plus bevacizumab. The median PFS was 3.9 and 3.5 months, respectively. The ORR was 22.2% and 2.2%, respectively, and the DCR was 62.2%. Quan et al. [25] found that 40 patients with NS-NSCLC who received bevacizumab-containing chemotherapy achieved a 5% response rate and 50% DCR after 2 cycles, with a median OS of 29.6 months. The results of this study showed that the median PFS of the treatment group and the control group was 6.2 months (95% CI: 5.86–6.56 months) and 5.1 months (95% CI: 4.956–5.191 months), respectively. The median OS of the observation group and the control group was 14.4 months (95% CI: 12.681–16.113 months) and 10.6 months (95% CI: 8.759–12.391 months), respectively, and the difference was statistically significant (*P* < 0.05). It is suggested that, based on chemotherapy, local addition of bevacizumab can achieve local and systemic control of the disease and can bring significant therapeutic benefits for second-line treatment of NS-NSCLC patients with negative driver genes. The synergistic effect of bevacizumab and chemotherapeutics may be due to the mechanism of bevacizumab in inducing normalization of tumor blood vessels, thereby improving the hypertensive state of tumor interstitial and promoting the effective distribution of chemotherapeutics into tumor tissues [26].

The results of this study showed that patients with low VEGF expression, pleural effusion pH ≥ 7.35, and high CD4^+^/CD8^+^ ratio in pleural effusion were more effective in treatment and had a longer survival period. According to reports [26, 27], the pH value of MPE was related to the survival rate. The results of the study by Sahn et al. also showed that patients with a MPE pH value less than 7.30 had a shorter survival period than patients with a pH value ≥7.30 [21]. Rodriguez-Panadero and colleagues observed that low MPE pH was associated with survival and the degree of intrapleural tumor accumulation. The relationship between MPF, pH, and survival rate may be due to extensive tumor deposition leading to the accumulation of glycolysis end products in the pleural cavity, which indicates that advanced or aggressive malignancies have poor survival rates [28]. VEGF is a glycoprotein that is specific to endothelial cells and stimulates angiogenesis. In addition to stimulating the formation of new blood vessels, VEGF can also promote vascular permeability, and MPE is related to high levels of VEGF in serum, plasma, and MPE [29]. VEGF plays an important role in the development of MPE. Cancer cells use VEGF-related mechanisms to invade or metastasize to the pleural cavity. Therefore, cancer cells can promote high levels of VEGF in the pleural cavity, thereby inducing the formation and accumulation of effusion [30]. It was reported that the high immunohistochemical expression of VEGF and high levels of VEGF in serum and pleural effusion before treatment may be independent predictors of poor survival in patients with advanced NSCLC [31]. This result is consistent with our research results. Studies have shown that if CD4^+^*T* cells were dominant in MPE, the proportion of CD8^+^*T* cells was significantly lower than that of CD4^+^*T* cells [32]. On the contrary, in the pleural cavity of patients with lung cancer without MPE, the number of CD4^+^*T* cells was significantly lower than that of CD8^+^ T cells [33]. For *T* cells to kill tumor cells, antigen-presenting cells must first present the surface antigens of tumor cells to T cells. T cells perform their function to eliminate tumor cells only after they were activated. However, studies have found that antigen-presenting cells, including dendritic cells with the strongest presenting ability, are defective in the presentation of tumor antigens, which frees tumor cells from sanction, that is, “immune escape.” The related mechanism is that tumor cells release some factors, such as VEGF, that prevent the maturation of dendritic cells, thereby preventing dendritic cells from contacting T cells and cutting off the process of antigen presentation. Therefore, Bevacizumab can restore tumor antigen presentation by dendritic cells and activate T cells [34]. In this study, we use bevacizumab to inhibit VEGF and increase the ratio of CD4^+^T lymphocytes in the effusion, which may be related to the local recruitment of CD4^+^*T* cells to exert antitumor effects.

## 5. Conclusions

In conclusion, the applications of albumin paclitaxel + bevacizumab and intrapleural perfusion of lobaplatin are effective, and the adverse reactions are tolerable in patients with driver gene-negative NS-NSCLC complicated with malignant pleural effusion. The VEGF and pH of pleural effusion before treatment are independent poor prognostic factors, and the change in the ratio of CD4+/CD8^+^ in pleural effusion before and after treatment is an independent predictor of treatment efficacy. Combined bevacizumab treatment could reverse the adverse factors to a certain extent. This low-dose combination treatment regimen is worthy of clinical promotion.

## Figures and Tables

**Table 1:** Comparison of clinical data between the two groups of patients.

Characteristics	Treatment group (*n* = 62)	Control group (*n* = 64)	*χ* ^*2*^	*P*
Gender			0.002	0.968
Male	38	39		
Female	24	25		
Age (years)			0.022	0.881
<60	36	38		
≥60	26	26		
Stage			0.166	0.920
M1a	49	51		
M1b	8	7		
M1c	5	6		
MPE characteristic			0.001	0.97
Bloody	25	26		
Non-bloody	37	38		
VEGF expression			0.0003	0.99
High	28	29		
Low	34	35		
Liquor pleurae pH			0.001	0.97
<7.35	25	26		
≥7.35	37	38		
Hydrothorax CD4^+^/CD8^+^ value			0.0004	0.10
High	30	31		
Low	32	33		

**Table 2:** Comparison of clinical efficacy between the two groups of patients.

Group	CR	PR	SD	PD	ORR (%)	DCR (%)
Treatment group	4	28	25	5	32 (51.6)	57 (91.9)
Control group	0	20	26	18	20 (31.3)	46 (71.9)
*χ* ^*2*^					5.39	8.49
*P*					0.02	0.004

**Table 3:** Comparison of adverse reactions between the two groups of patients.

Adverse reactions	Treatment group (*n* = 62)		Control group (*n* = 64)		*χ* ^*2*^	*P*
	1-2	3-4	1-2	3-4		
Hypertension	5	1	4	0	0.506	0.477
Weakness	12	1	6	10	0.289	0.591
Nausea/vomiting	9	3	8	6	0.122	0.727
Proteinuria	4	5	6	2	0.109	0.741
Neutropenia	1	3	2	0	0.768	0.381
Thrombocytopenia	2	6	5	1	0.397	0.529
Anemia	8	2	6	8	0.674	0.412
Peripheral neurotoxicity	25	5	22	4	0.768	0.381

**Table 4:** Comparison of the quality of life scores between the two groups of patients ($\bar{x}\pm s$, points).

Scores		Treatment group (*n* = 62)	Control group (*n* = 64)	*t*	*P*
GH	Before	60.13 ± 4.36	59.56 ± 5.25	0.662	0.509
	After	66.25 ± 6.28^*∗*^	60.26 ± 7.16^#^	4.986	<0.001
RP	Before	45.46 ± 5.08	46.14 ± 5.12	0.748	0.456
	After	58.28 ± 6.02^*∗*^	51.04 ± 6.16^*∗*^	6.670	<0.001
BP	Before	58.73 ± 4.76	59.01 ± 4.98	0.322	0.748
	After	68.08 ± 5.68^*∗*^	62.25 ± 6.11^*∗*^	5.543	<0.001
SF	Before	66.78 ± 7.14	65.05 ± 7.05	1.368	0.174
	After	73.28 ± 6.58^*∗*^	67.68 ± 6.08^#^	4.964	<0.001
VT	Before	51.68 ± 6.06	52.28 ± 6.36	0.542	0.589
	After	63.28 ± 6.88^*∗*^	52.68 ± 6.62^#^	8.814	<0.001

Compared with that before treatment of the group, ^*∗*^*P* < 0.05, #*P* < 0.05.

**Table 5:** Univariate analysis of factors affecting the prognosis of patients in the treatment group.

Characters	PFS (months)	95% CI	*P*	OS (months)	95% CI	*P*
VEGF expression			<0.001			<0.001
High	4.901	4.712–5.090		12.309	11.615–13.002	
Low	7.146	6.483–7.809		16.749	16.450–17.048	
Liquor pleurae pH			<0.001			<0.001
＜7.35	5.071	4.678–5.463		13.022	12.623–13.420	
≥7.35	6.678	6.237–7.119		17.054	16.748–17.361	
Hydrothorax CD4+/CD8+ value			0.002			<0.001
High	6.594	6.324–6.864		16.349	15.886–16.833	
Low	5.788	5.307–6.268		11.884	11.592–12.176	

## Data Availability

The datasets used to support the findings of this article are available from the corresponding author upon reasonable request.

## Supplementary Materials

Supplementary Materials CONSORT 2010 checklist.
